# Annotations

**Published:** 1892-11-19

**Authors:** 


### ANNOTATIONS.

**A Horses' Hospital.** If horses had been men, and men horses, it can hardly be doubted that there would have been more than one horses' hospital before the last decade of the nineteenth century. The horse is of a gentler nature than man, and would not have allowed a faithful servant to be so long neglected when sick. Mr. John Atkinson, F.R.C.V.S., advocates in the daily newspapers the founding of an East London branch of the Horses' Hospital, with out and in-patient departments, where help may be obtained free for horses by all those poor men, costers and others, who would willingly give their faithful animals medical treatment if they had the wherewithal to pay for it. It is one of the sorrows of London that we are obliged to use our horses so sternly. The horses that come to the metropolis may bid farewell to ease, and even to hope. They come to be used, and they are used, most strenuously and unceasingly. If they are well they can bear it; but if they are sick, or lame, or injured, they must still work, unless they are so very bad as to come within the reach of the protective law. The sufferings of London's ten times ten thousand horses in a single day would make one sicken with horror and amazement if we could but realise them. Alas, poor dumb brutes! And nothing is done for them. But we must try now to make a beginning. Mr. John Atkinson deserves the thanks of us all in that he has brought the subject into notice. This is an "everybody's question;" a question for those who use horses, and for those who, though seldom using, yet love them. It is a question which two classes of persons can rapidly bring to a head: the two classes are newspaper writers and men of wealth. Will the newspapers become voices for the helpless dumb, and will the rich listen to the "voiceless cry of the horse"? Let us hope that neither sympathy nor help will be long delayed.

**Is Science too Egotistical?** At the opening of the Oxford Medical Society the other day Sir James Paget was the orator. In the course of an address full of serviceable philosophy and honest English feeling, Sir James spoke as follows: "He ventured to think that the scientific man would not be altogether discredited if, with the help of those in practice, some of his results might be turned into immediate utility." This is a very innocent-looking sentence, but to the initiated it means a good deal. Science is now essaying to take the absolute lead in all human affairs. It is very desirable that we should thoroughly understand our new leader and see what sort of a creature she—for science for some unaccountable reason prefers to be of the feminine gender—really is. One asks of a leader that he be full of generosity; that he know not only how to choose his followers, but how to distinguish and to reward them. Above all one asks that he lead, not for his own profit or glorification, but for the advancement and honour of the whole body of which he is the leader. How does science come out when tested by these conditions? What is her attitude towards the great half-cultured world which she is so ambitious to lead? Sir James Paget's remark is a key which opens the door upon one of life's chambers, which is illuminated by a somewhat baleful light. Many men of science profess a magnificent contempt for practice. They desire the honours of leadership, but they repudiate its obligations. For those men and women who are unlearned in science, they affect, and often really feel, a good deal of contempt. That is not the way to lead, at any rate, the English people. They have refused to be dragooned by kings and conquerors, and they certainly will not be "lorded over" by men of science. There is only one attitude which science can rationally take up, and that is the attitude of teaching all the world and helping all the world to become as scientific as herself. A very great leader of the people, who has been an acknowledged leader for hundreds of years, had a very humble class of men and women for his immediate personal followers, and yet he addressed those humble persons in these terms: "All things are yours, whether the world, or life, or death." That is the true spirit of leadership. The principles that are embodied in such a leader are bound to prevail. Sir James Paget administered a quiet, but not the less significant, rebuke to those scientists who scorn practice and despise the unscientific. Let us hope they will have intelligence enough to understand it, and profit by it.

**The Value of R.I.B.A.** Mr. Hugh Alexander, who has been appointed Chairman of the Sanitary Inspectors' Association for the sixth year in succession, was very severe upon architects in his inaugural address last Saturday, and apparently with good reason. "Workmen's Dwellings"—the subject of Mr. Alexander's discourse—are to be looked at from two points of view: viz., the point of view of the workmen who inhabit them, and the point of view of the other inhabitants of the town in which they are placed, and in which, if they are unwholesome, they may become centres of epidemic disease, and deadly pestilence. Mr. Alexander informed his audience that country people have the power to insist upon the erection of buildings which shall be healthy for working men, but that they are too stupid to use it. London people, on the other hand, have not even the power, and they in their turn are too stupid to ask Parliament for it. Under these circumstances the actual condition of workmen's dwellings in some parts of London and other large towns is as delightfully dangerous to the public safety as the most ignorant of rich property holders or foolish citizens could possibly desire. For much of this deadly unwholesomeness, Mr. Alexander thinks architects are chiefly to blame. He seems to consider that they are among the very silliest of civilized people. Whilst wholesomeness is one of the most necessary conditions of all house-building, and ornamentation is by comparison a mere trifle, the average architect devotes his whole mind to ornament, but to wholesomeness he does not appear to give so much as a single thought. This is turning the pyramid upon its apex with a vengeance. It is as if the doctor should prescribe the hot room of a Turkish bath for a patient down with typhoid fever, or the parson should recommend a course of peculation from the till for the cure of the vice of gambling. Quoting the words of the report of a recent Royal Commission, Mr. Alexander says: "Many of the so-called 'model blocks' of dwelling-houses are built on the flat system. To say that some of these model blocks are built without due regard to sanitary requirements would be a misuse of language. The fact is that they are built in gross violation of the very first principles of sanitation. The dwellings contain rooms, many of which can never be penetrated by the rays of the sun. These dark and gloomy habitations are, in our opinion, far more likely to become a source of danger to the public health than are even the worst of the dilapidated cottages to which public attention has ever been drawn by the Mansion House Council." Such is the language of a Royal Commission. Mr. Alexander, looking about him for the real culprit, puts the saddle on the right horse, and that horse is the architect. "It could be proved," says he, "that some of the very worst of these so-called 'model dwellings' had been planned by men who wrote R.I.B.A. after their name." Mr. Alexander suggests the drastic measure of striking the names of such planners of dwelling-houses from the roll of British architects. A common-sense public will be very much disposed to agree with him.

